# Exploring the associations between adverse childhood experiences (ACEs) and adolescent cancer risk behaviours in the ALSPAC cohort

**DOI:** 10.1186/s12889-023-17516-4

**Published:** 2024-01-05

**Authors:** Paul Okediji, David Troy, Jon Heron, Ruth R. Kipping, Richard M. Martin, Caroline Wright

**Affiliations:** 1 Population Health Sciences, Bristol Medical School, University of Bristol, Bristol, UK; 2 MRC Integrative Epidemiology Unit (IEU), Population Health Sciences, Bristol Medical School, University of Bristol, Bristol, UK; 3 NIHR Bristol Biomedical Research Centre, University Hospitals Bristol and Weston NHS Foundation Trust and the University of Bristol, Bristol, UK

**Keywords:** Cancer risk behaviours, Adverse childhood experiences, ACE, Adolescence, ALSPAC, UK birth cohort

## Abstract

**Background:**

Some modifiable risk factors for cancer originate during adolescence. While there is evidence indicating relationships between adverse childhood experiences and health risk behaviours generally, little is known about how childhood adversity influences the engagement of adolescents in cancer risk behaviours. This study aimed to determine the relationship between adverse childhood experiences and adolescent cancer risk behaviours.

**Methods:**

Data were collected prospectively from birth to age 18 years on children born to mothers enrolled into the Avon Longitudinal Study of Parents and Children (ALSPAC) cohort study. Multivariable linear regression models assessed relationships of a composite exposure measure comprised of adverse childhood experiences (total number of childhood adversities experienced from early infancy until age 9 years) with multiple cancer risk behaviours. The latter was expressed as a single continuous score for tobacco smoking, alcohol consumption, obesity, unsafe sex, and physical inactivity, at ages 11, 14, 16 and 18 years. Analysis was carried out on the complete case and imputation samples of 1,368 and 7,358 participants respectively.

**Results:**

All adolescent cancer risk behaviours increased in prevalence as the adolescents grew older, except for obesity. Each additional adverse childhood experience was associated with a 0.25 unit increase in adolescent cancer risk behaviour (95% CI 0.16–0.34; *p* < 0.001). Individually, parental substance misuse (β 0.64, 95% CI 0.25–1.03, *p* < 0.001) and parental separation (β 0.56, 95% CI 0.27–0.86, *p* < 0.001) demonstrated the strongest evidence of association with engagement in adolescent cancer risk behaviour.

**Conclusion:**

Childhood adversity was associated with a greater degree of engagement in adolescent cancer risk behaviours. This finding demonstrates the need for targeted primary and secondary prevention interventions that reduce engagement across multiple cancer risk behaviours for children and adolescents who have experienced adversity in childhood, such as parental substance misuse and separation, and reduce exposure to adversity.

**Supplementary Information:**

The online version contains supplementary material available at 10.1186/s12889-023-17516-4.

## Background

The United Kingdom (UK) had 458,000 incidents of cancers in 2020 with a ranking of 9th in the world for cancer incidence globally [1]. Around 180,000 people die in the UK annually from cancer-related causes [1], accounting for ~ 25% of all deaths [2]. The impact of premature mortality associated highlights the need for cancer prevention and control [3]. Cancer aetiology is multifactorial, and cancer risk is increased by exposure to a broad range of potentially modifiable risk factors that offers an opportunity to prevent cancers through targeted interventions [4]. Cancer Research UK estimate that ~ 40% of all cancers can be prevented by reducing exposure to tobacco smoking, excessive consumption of alcohol, physical inactivity, high body mass index, poor diet, excess sun exposure and unsafe sex [5, 6].

Many of these behaviours originate during adolescence [7–9], a critical phase of life characterised by physical, social, cognitive and emotional changes that prepare the adolescent for adult life [4]. From a life-course perspective, adolescence presents a period of vulnerability in which habits formed during that time can persist into adulthood, including cancer risk behaviours [10]. By age 15 years, up to 40% of adolescents have smoked tobacco at least once, 40% have been drunk at least twice, and 30% have had sexual intercourse in England [11]. 80% of adult smokers initiate smoking before age 18 years [10, 12].

Several theories attempt to explain the propensity of adolescents to engage in health-risk behaviours. Gateway theories argue that engagement in one behaviour increases the likelihood of engagement in other risk behaviours through a reduction in the perceived danger inherent in such behaviours and an increased desire to try out other risk behaviours [13]. The “problem behaviour theory” also explains why adolescents are likely to engage in risk behaviours as a way of showing their disregard for conventionality and expressing their independence and maturity [8]. A number of studies have pointed at the role of childhood adversities, also referred to as adverse childhood experiences, in the engagement of individuals in health-damaging behaviours such as harmful alcohol consumption, smoking and drug use [14, 15]. It has been suggested that the effect of childhood adversities on health risk behaviours might be additive (where the combined effect of two childhood adversities equals the sum of the effects of each childhood adversity acting independently) leading to an increased propensity for engagement in health risk behaviours [16]. While there is some evidence pointing at relationships between adverse childhood experiences and health risk behaviours generally, little is known about how childhood adversities influence adolescents’ specific engagement in cancer risk behaviours.

Previous analyses using data from the Avon Longitudinal Study of Parents and Children (ALSPAC) database have explored the associations between adolescent (age 11–18 years) and young adult (age ~ 24 years) cancer risk behaviours (tobacco smoking, alcohol consumption, obesity, sexual risk and physical inactivity) [17]. Distinct groups of adolescents characterised by consistently high- and low-cancer risk behaviours during adolescence were identified, with associations of large magnitude between adolescent and early adult cancer risk behaviours [17]. Building on this body of work, the current study aimed to assess the relationship between adverse childhood experiences and adolescent cancer risk behaviours using a UK birth cohort, and focusing on the hypothesis that adverse childhood experiences are associated with adolescent engagement in cancer risk behaviours.

## Methods

### Study design and setting

Using a prospective cohort study design, data used in this study were drawn from ALSPAC, an ongoing prospective observational population-based birth cohort study investigating the effects of a wide range of influences on health and development across the life course [18, 19]. Pregnant women, resident in Avon, UK and with expected dates of delivery from 1st April 1991 to 31st December 1992 were invited to take part in the study. The initial number of pregnancies enrolled was 14,541 (for these at least one questionnaire has been returned or a “Children in Focus” clinic had been attended by 19/07/99). Of these initial pregnancies, there was a total of 14,676 foetuses, resulting in 14,062 live births and 13,988 children who were alive at 1 year of age. Details of all available questionnaires and data can be found through a searchable data dictionary (http://www.bristol.ac.uk/alspac/researchers/our-data/). Ethical approval for the study was obtained from the ALSPAC Law and Ethics Committee and local Research Ethics Committees. Informed consent for the use of data collected via questionnaires and clinics was obtained from participants following the recommendations of the ALSPAC Ethics and Law Committee at the time.

### Exposure measure

Adverse childhood experiences were selected as the main exposures. A child is said to have experienced an adverse childhood event if they meet the criteria (as defined in Supplementary Material Section A-1) for at least one type of adverse childhood experience by age 9 years [20]. Adversities include physical, sexual and emotional abuse; parental mental health problems or suicide, criminal convictions, substance misuse; and child bullying, parental separation and/or violence between parents. The definitions of each adverse childhood experience used in this study has been described by Troy et al. [20] and the approach with which each adversity has been derived is described in the supplementary material (Supplementary Material Section A-1). Data were collected on these adverse childhood experiences from early infancy until age 9 years (used as a cut-off to delineate interval between exposure and outcomes with a lag period of two years in between) using self-administered questionnaires directed at mothers and partners (about exposures to the nine childhood adversities except for child bullying that was based on data obtained directly from the child). A cumulative adversity exposure measure was derived by adding up the number of childhood adversities each adolescent experienced as a total continuous score.

### Outcome measure

As detailed in Wright et al. [17], repeated measures of adolescent cancer risk behaviours – tobacco smoking, alcohol consumption, obesity, unsafe sex and physical inactivity – measured by questionnaires at ages 11, 14, 16, and 18 years, were used for the derivation of the outcome variable. Each cancer risk behaviour at each time point was coded as a binary indicator with participants scoring “1” if the risk was present or “0” otherwise [17]. Engagement in cancer risk behaviours across adolescence was then summarised as a single continuous measure using the approach more commonly adopted for tobacco use when expressing total exposure in terms of “pack-years”. The scale was derived by summing the binary indicators into a total for each time point (11, 14, 16, and 18 years) and then combining these four totals into a single scale to describe total engagement in cancer risk behaviours across the adolescent period. This yielded a continuous, roughly symmetric scale which took values from zero to 21 within the observed sample. The outcome, which we refer to as “Area Under the Curve” given its method of derivation, can be interpreted as measuring CRB-years of behaviour, and therefore a regression parameter of 0.5 can be taken to represent the engagement in one risk behaviour for an additional 6-months. More details can be found in Supplementary Material SA-2.

### Confounder measures

Variables that are plausible causes of both the exposures and outcomes of interest were considered as confounders, and included male/female sex, parental social class, household income, housing tenure, maternal education, and maternal age at delivery. Apart from child sex, these data were collected by postal questionnaires submitted to the main carer during the antenatal and early postnatal period.

*Sex*: Adolescent’s biological sex recorded at birth; *parental social class*: measured using parent’s highest social class (professional; managerial and technical; skilled non-manual; and skilled manual, part or unskilled manual); *mother’s education*: This assesses the mother’s highest level of education and classifies as “less than O-level/GCSE (i.e. no high-school qualifications)”, “qualifications up to high-school level”, and “beyond high-school, including undergraduate and postgraduate degrees”; *household income*: Average amount of money each household earns, split into quintiles of high to low income; *housing tenure during pregnancy*: Assesses type of home ownership by parents of the adolescents in the study, and categorized as mortgaged or own property, privately rented property, or subsidized rental property; *mother’s age*: Measured as mother’s age at time of pregnancy.

### Statistical analysis

All relevant data for the study were collected at different time intervals using self-administered questionnaires issued during clinic visits, and responses to postal questionnaires provided by parents/caregivers. There were complete data on exposure, outcome, and confounders for 1,368 participants (9.8% of ALSPAC participants), referred to as the complete case sample. Supplementary Material Section B contains further details the sample definition including a flowchart and subsample comparisons. The complete case sample was not representative of the overall ALSPAC sample as participants in these complete case samples were more likely to be females, in higher social classes, live in mortgaged/owned property, and have education above O-level (Supplementary Table S4).

Descriptive analyses were conducted to summarise the prevalence of adolescent cancer risk behaviours in the study population. Tetrachoric correlations, which measure correlations between two binary variables, were carried out between pairs of cancer risk behaviours at ages 11, 14, 16 and 18 years. Univariable and multivariable linear regression models were used to analyse the prospective relationship between adverse childhood experiences (both individual adverse childhood experiences and combined exposure measure) and adolescent cancer risk behaviour. The multivariable linear regression model was adjusted for sex, social class, mother's education, household income, housing tenure, and mother's age.

#### Missing data

Multiple imputation by chained equations was used to account for missing data [21]. Missing data on cancer risk behaviours, childhood adversities, and socioeconomic variables were imputed to create the final imputed sample (*N* = 7,358). Imputation of each of the study variables used a bespoke combination of auxiliary variables (that were not included in the analysis model but provide additional information about the missing values), including alternative socioeconomic variables. A number of data sets with a varying number of imputations were generated using the ice command in Stata/MP v15.1 [22]. Monte Carlo errors were used to compare the probability that imputation would be reproducible. The imputation of 100 and 250 data sets using the rules of thumb by White et al. [23] were compared, and 250 was chosen as the most appropriate number of imputed data sets as it provides a sufficient level of reproducibility. The main results reported are based on the imputed dataset, with the complete-case estimates shown in the Supplementary Material.

## Results

**Fig. 1:**
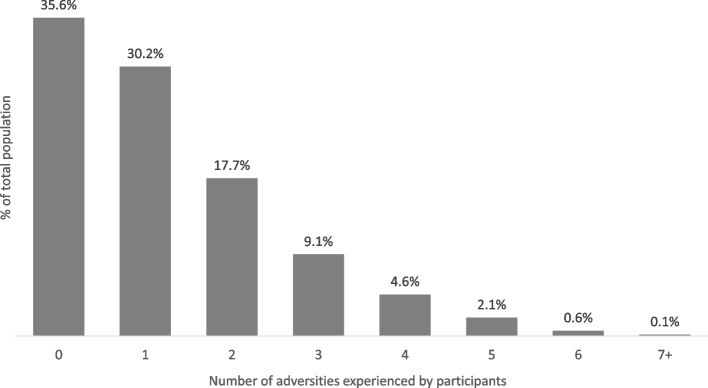
Distribution of the study participants by the number of adversities they experienced during childhood (*N* = 7,358, imputed sample)

The distribution of the study population in the imputation and complete case samples, on the basis of exposure and outcome variables, is described in Table 1. The most common adversity the adolescents experienced was parental mental health problems or suicide (37.7% of the adolescents). Parental separation (19.4%), violence between parents (18.5%), and emotional abuse (16.8%) were also common among the adolescents. The average number of adverse childhood experiences by the adolescents was 1.3 (SE = 0.016) with most participants experiencing no ACE (35.6%) and another 30.2% experienced one ACE (Fig. 1). Overall, 7.4% of the adolescents had experienced four or more adversities. Sexual abuse had the lowest prevalence rate of 0.5%. Compared with the complete case sample, prevalence rates of the various adverse childhood experiences were slightly higher in the imputation sample except in the case of physical abuse where the prevalence rates in both samples were generally the same.

**Table 1:** Outcome and exposure statistics by imputed and complete case samples

Variables	All available sample (max = 7,358)		Complete case sample *N* = 1,368	Imputation data *N* = 7,358
	**Total N**	**Mean / N (%)**	**Mean / N (%)**	**Mean / N (%)**
**Continuous outcome measure**				
Cancer risk behaviour; *Mean (SD/SE)*	1,786	9.51 (SD = 4.20)	9.44 (SD = 4.17)	9.71 (SE = 0.067)
**Continuous exposure measure**				
Adverse childhood exposures as a combined measure*; Mean (SD/SE)*	4,255	1.16 (SD = 1.29)	1.01 (SD = 1.19)	1.26 (SE = 0.016)
**Binary exposure measures**				
Sexual abuse	7,307	36 (0.5%)	5 (0.4%)	0.5% (SE = 0.08)
Emotional abuse	6,795	1,138 (16.8%)	200 (14.6%)	16.8% (SE = 0.46)
Physical abuse	7,356	470 (6.4%)	88 (6.4%)	6.4% (SE = 0.29)
Bullying	5,331	601 (11.3%)	137 (10.0%)	11.5% (SE = 0.44)
Violence between parents	6,133	1,118 (18.2%)	210 (15.4%)	18.5% SE = (0.50)
Parental substance use	7,316	663 (9.1%)	93 (6.8%)	9.1% (SE = 0.34)
Parents’ mental health problems or suicide	7,347	2,770 (37.7%)	428 (31.3%)	37.7% (SE = 0.57)
Parent conviction	7,333	461 (6.3%)	76 (5.6%)	6.3% (SE = 0.28)
Parental separation	7,233	1,400 (19.4%)	146 (10.7%)	19.4% (SE = 0.47)

SD (Standard Deviation) describes spread of values in the sample for continuous measures

SE (Standard Error) describes precision of summary statistic (derived using Rubin’s rules)

**Fig. 2:**
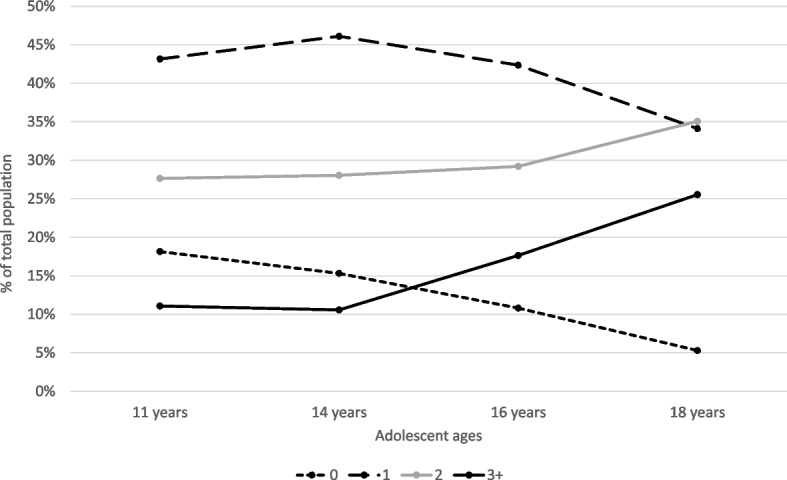
Engagement in of cancer risk behaviour during adolescence (*N* = 7,358, imputed sample). Lines indicate the number of cancer risk behaviours reported

Across all five cancer risk behaviours investigated, there was a general increase in concurrent engagement in two or more cancer risk behaviours while the proportion of adolescents who engaged in zero cancer risk behaviours reduced as they grew older (Fig. 2). Assessing relationships between cancer risk behaviours, modest positive correlations between alcohol consumption and tobacco smoking (0.35 ≤ r ≤ 0.63) were observed between ages 11 and 18 years. At age 11, there was some correlation between physical inactivity and obesity (*r* = 0.20), which weakened progressively through ages 14 (*r* = 0.13) and 16 (*r* = 0.01) years. At ages 14, 16 and 18 years, there were modest correlations between unsafe sex and tobacco smoking (0.32 ≤ r ≤ 0.36).

There was strong evidence of a positive association between childhood adversity and multiple adolescent cancer risk behaviours (Table 2). Analyses of the imputed data show that each additional adversity a child experiences is associated with a 0.25 increase in the number of cancer risk behaviours (95% CI 0.16 – 0.34; *p* < 0.001). Emotional abuse, substance use, violence between parents, mental health problems in parents and parental separation demonstrated strong evidence of association with multiple cancer risk behaviours in adolescence (*p* < 0.05, Table 2). Among all the childhood adversities, the use of prohibited substances by parents was most strongly associated with adolescent cancer risk behaviour with a 0.64 unit increase in cancer risk behaviour (95% CI 0.25 – 1.03, *p* = 0.001). Similarly, parental separation was associated with an increased risk as it is associated with an increase of 0.56 in the number of cancer risk behaviours (95% CI 0.27 – 0.86, *p* < 0.001). Comparing the results of the regression analysis using the imputed data (Table 2) with those using the complete case sample (see Supplementary Table S5), the findings were mostly similar with slight differences in the magnitude of association for adverse childhood experiences combined variable, substance use, and parental separation.

**Table 2:** Linear regression analyses showing associations between childhood adversity and adolescent cancer risk behaviour *(N* = *7,358; imputed sample)*

Childhood adversity variable	Univariable regression models			Multivariable^a^ regression models		
	**β**	**95% CI**	*p*	**β**	**95% CI**	*p*
Adverse childhood experience total score (per additional ACE)	0.28	0.19 ‒ 0.36	< 0.001	0.25	0.16 ‒ 0.34	< 0.001
Physical abuse	0.31	-0.14 ‒ 0.76	0.179	0.36	-0.09 ‒ 0.81	0.113
Sexual abuse	1.14	-0.50 ‒ 2.78	0.173	0.74	-0.88 ‒ 2.36	0.373
Emotional abuse	0.51	0.19 ‒ 0.82	0.002	0.51	0.20 ‒ 0.82	0.001
Bullying	0.24	-0.12 ‒ 0.60	0.194	0.22	-0.13 ‒ 0.58	0.220
Violence between parents	0.45	0.14 ‒ 0.75	0.004	0.38	0.08 ‒ 0.68	0.012
Parental substance use	0.71	0.33 ‒ 1.10	< 0.001	0.64	0.25 ‒ 1.03	0.001
Parents’ mental health problems or suicide	0.49	0.25 ‒ 0.72	< 0.001	0.42	0.18 ‒ 0.65	< 0.001
Parent conviction	0.35	-0.11 ‒ 0.81	0.140	0.28	-0.18 ‒ 0.74	0.232
Parental separation	0.71	0.42 ‒ 1.00	< 0.001	0.56	0.27 ‒ 0.86	< 0.001

^a^ Regression models between each individual adversity variable and cancer risk behaviour controlled for sex, social class, mother's education, household income, housing tenure, and mother's age

## Discussion

This study investigated relationships between childhood adversities and adolescent cancer risk behaviours. Overall, the prevalence of cancer risk behaviours increased as the adolescents grew older with more adolescents engaging in two or more cancer risk behaviours concurrently from age 14 up until age 18. The clustering of risk behaviours in adolescence has been well documented in literature, with significant implications on health and social outcomes in early adulthood [4, 24, 25]. Increasing engagement in cancer risk behaviour through adolescence was also reflected in the individual behaviours whose prevalence increased with age except for obesity that remained generally the same with a marginal decline between ages 11 and 14 years. Additionally, this study found strong evidence of associations between unsafe sex and each of tobacco smoking and alcohol consumption, especially at ages 16 and 18. These three behaviours all trigger the dopaminergic system associated with dependence and addiction [26]. Furthermore, they are adult-like behaviours which adolescents feel the need to engage in to be accepted into the “adult society” and they are associated with disinhibition and sensation seeking [26].

There was strong evidence of a positive association between childhood adversity and adolescent cancer risk behaviours. A previous cross-sectional study suggested a link between adversities and multiple risk behaviours among British adolescents [20]. Our study confirms a similar association between adversities and behaviours specific to cancers assessed longitudinally. Across the specific adversities investigated, parental substance abuse had the largest coefficient of effect on adolescent multiple cancer risk behaviours, followed by parental separation. The exact mechanism explaining the relationship between parental substance use and multiple cancer risk behaviours during adolescence is not clear as there is a complex web of psycho-social factors involved which can be difficult to untangle [27].

However, there is evidence that parents have a huge influence on children's health behaviours especially considering their roles as models, monitoring their children's behaviours, and reinforcing positive behaviours [28]. When a parent engages in substance use, there is a high tendency that this behaviour becomes ‘normalised’ in a way that the child sees it as acceptable to engage in similar behaviours. A parent’s ability to provide necessary monitoring and guidance to the child often becomes impaired when under the influence of substances [27]. There is also evidence indicating that parents with substance use disorders are about three times more likely to abuse their child physically or sexually, thereby compounding a cycle of neglect [29].

Data from this study showed that parental separation is associated with an increase in engagement in cancer risk behaviours during adolescence. This correlates with a previous study reporting that parent substance use have strong effects on multiple risk behaviours [20]. Parental separation is often associated with conflict which in itself is a risk factor for problem behaviours in children, indicating that conflict may be a confounder in the relationship between parental separation and cancer risk behaviours [30]. Furthermore, parents who have separated are less likely to provide the necessary parental monitoring required to mould their children's behaviours. According to DiClemente, adolescents with less parental monitoring have greater odds of alcohol consumption, marijuana use, unsafe sex, and being arrested [31].

Our study found that 7.4% of the study population had experienced four or more adverse childhood experiences, which is similar to a national household survey in England which reported that 9% of the English population fell in the same category [32]. This study also shows that as the number of adversities increases, the probability of engaging in cancer risk behaviours during adolescence rises. This is consistent with the cumulative risk theory and previous studies that have documented the dose–response effect of an increasing number of adverse childhood experiences on engagement in health-risk behaviours in later life [15, 33]. There is evidence showing that childhood adversity is associated with emotional dysregulation and blunted reward responsivity, which in turn increases the propensity for alcohol consumption, tobacco smoking, and unsafe sexual behaviour in a continuous positive feedback loop [34]. Expectedly, a British birth cohort study has shown that women who had two or more adversities had a twofold risk of developing cancer before 50 years [35].

This study is one of the first to investigate the relationship between early life adversities and adolescent cancer risk behaviour. It uses a longitudinal approach which provides a moderate life course perspective that takes distant exposures into consideration in the investigation of current behavioural outcomes. Adjustment for all potential confounders reduced the risk of residual confounding which could have reduced the reliability of the findings; even though there was little difference recorded between the results of adjusted and unadjusted models.

One of the potential limitations of this study is the use of self-reports for data collection which is associated with social desirability bias. Nonetheless, with repeated data collection in the ALSPAC study, participants are expected to have developed confidence in their anonymity which might reduce the influence of bias. As described by Houtepen et al., data on childhood adversity was collected mostly from parents which increases the risk of potential misclassification and bias [36]. However, many of the ACE prevalence estimates in this study are comparable to what is obtained in other studies, including a national household survey, suggesting minimal effects on the results [32, 36].

Secondly, the results of this study need to be generalised with caution as the ALSPAC database is not a nationally representative sample. Also, there were differences in the adverse childhood experiences prevalence estimates between the complete case and imputed case analyses, with estimates for the complete case sample smaller than those recorded using imputed data. This observation is probably due to loss to follow-up which tends to be socially patterned, meaning that less adolescents with adversities would be found in the complete case sample. The large confidence intervals observed for the association between individual adverse childhood experiences and multiple cancer risk behaviours further suggest that the results of the regression analysis need to be interpreted with caution in the light of the small sample size particularly in the complete case sample.

Finally, there was a large amount of missing data in follow-up data collection. This can reduce statistical power and potentially introduce bias. For bias to be introduced however, the outcome measure has to be conditionally related to whether the participants remain in the sample at time of data collection [37]. As in many other similar studies based off the ALSPAC database [4, 17], this study assumes that the data is missing at random. More work will be required to untangle the association between physical inactivity and obesity, with emphasis on the role of diet and other potential risk factors of obesity among adolescents. It will also be interesting to note the point of initiation of these cancer risk behaviours to further inform public health interventions aimed at preventing the initiation of cancer risk behaviours in children and adolescents.

## Conclusion

The association of cumulative childhood adversities with future adolescent engagement in multiple cancer risk behaviours demonstrates the need for targeted primary and secondary prevention interventions that can reduce engagement across multiple cancer risk behaviours for children and adolescents with adverse childhood experiences. With early initiation of many cancer risk behaviours, there is a need to determine how to target periods of vulnerability and intervening to reduce adversities in early childhood and thus preventing or delaying the start of cancer risk behaviours. The ultimate aim of such interventions would be to reduce cancer risk and prevent unnecessary cancer related morbidity and premature mortality.

## Supplementary Information

**Additional file 1.**

## Data Availability

The datasets analysed during the current study are available in the ALSPAC repository. The informed consent obtained from ALSPAC participants does not allow the data to be made freely available through any third party maintained public repository. However, data used for this submission can be made available on request to the ALSPAC Executive. The ALSPAC data management plan describes in detail the policy regarding data sharing, which is through a system of managed open access. Full instructions for applying for data access can be found here: http://www.bristol.ac.uk/alspac/researchers/access/. The ALSPAC study website contains details of all the data that are available (http://www.bristol.ac.uk/alspac/researchers/our-data/).

## Abbreviations

UK: United Kingdom
ALSPAC: Avon Longitudinal Study of Parents and Children
ACE: Adverse Childhood Experience

## References

[1] The Global Cancer Observatory (GLOBOCAN). United Kingdom. Lyon, France; 2020. https://gco.iarc.fr/today/data/factsheets/populations/826-united-kingdom-fact-sheets.pdf.

[2] Cancer Research UK. Cancer mortality statistics. https://www.cancerresearchuk.org/health-professional/cancer-statistics/mortality#heading-Zero. Published 2021. Accessed 30 Aug 2021.

[3] Hanly P, Ortega-Ortega M, Soerjomataram I. Cancer premature mortality costs in Europe in 2020: a comparison of the human capital approach and the friction cost approach. Curr Oncol. 2022;29(5):3552–64. 10.3390/curroncol29050287. 35621677 10.3390/curroncol29050287 PMC9139545

[4] Campbell R, Wright C, Hickman M, et al. Multiple risk behaviour in adolescence is associated with substantial adverse health and social outcomes in early adulthood: Findings from a prospective birth cohort study. Prev Med (Baltim). 2020;138:106157. 10.1016/j.ypmed.2020.106157 PMC7378566 32473267

[5] Gapstur SM, Drope JM, Jacobs EJ, et al. A blueprint for the primary prevention of cancer: targeting established, modifiable risk factors. CA Cancer J Clin. 2018;68(6):446–70. 30303518 10.3322/caac.21496

[6] Stein CJ, Colditz GA. Modifiable risk factors for cancer. Br J Cancer. 2004;90(2):299–303. 10.1038/sj.bjc.6601509. 14735167 10.1038/sj.bjc.6601509 PMC2410150

[7] Cancer Research UK. Statistics on preventable cancers. https://www.cancerresearchuk.org/health-professional/cancer-statistics/risk/preventable-cancers. Published 2015.

[8] Hale DR, Viner RM. The correlates and course of multiple health risk behaviour in adolescence. BMC Public Health. 2016;16(1):1–12. 10.1186/s12889-016-3120-z. 27246600 10.1186/s12889-016-3120-z PMC4888596

[9] World Health Organization. Adolescent and young adult health. Fact Sheets. https://www.who.int/news-room/fact-sheets/detail/adolescents-health-risks-and-solutions. Published 2021. Accessed 30 Aug 2021.

[10] Santelli JS, Sivaramakrishnan K, Edelstein ZR, Fried LP. Adolescent risk-taking, cancer risk, and life course approaches to prevention. J Adolesc Heal. 2013;52(5 SUPPL):S41–4. 10.1016/j.jadohealth.2013.02.017. 10.1016/j.jadohealth.2013.02.017 23601610

[11] Brooks FM, Magnusson J, Spencer N, Morgan A. Adolescent multiple risk behaviour: an asset approach to the role of family, school and community. J Public Health (Bangkok). 2012;34(SUPPL. 1):i48-56. 10.1093/pubmed/fds001. 10.1093/pubmed/fds001 22363031

[12] Rivara F, Park M, Irwin JC. Trends in adolescent and young adult morbidity and mortality. In: DiClemente R, Santelli J, Crosby R, editors. Adolescent Health: Understanding and Preventing Risk Behaviors. San Francisco, CA: Jossey-Bass; 2009. p. 7–31.

[13] Pudney S. The road to ruin? Sequences of initiation to drugs and crime in Britain. Econ J. 2003;113(486):C182–98.

[14] Bellis MA, Hughes K, Ford K, Ramos Rodriguez G, Sethi D, Passmore J. Life course health consequences and associated annual costs of adverse childhood experiences across Europe and North America: a systematic review and meta-analysis. Lancet Public Heal. 2019;4(10):e517–28. 10.1016/S2468-2667(19)30145-8. 10.1016/S2468-2667(19)30145-8 PMC7098477 31492648

[15] Campbell JA, Walker RJ, Egede LE. Associations between adverse childhood experiences, high-risk behaviors, and morbidity in adulthood. Am J Prev Med. 2016;50(3):344–52. 10.1016/j.amepre.2015.07.022. 26474668 10.1016/j.amepre.2015.07.022 PMC4762720

[16] Hughes K, Bellis MA, Hardcastle KA, et al. The effect of multiple adverse childhood experiences on health: a systematic review and meta-analysis. Lancet Public Heal. 2017;2(8):e356–66. 10.1016/S2468-2667(17)30118-4. 10.1016/S2468-2667(17)30118-4 29253477

[17] Wright C, Heron J, Kipping R, Hickman M, Campbell R, Martin RM. Young adult cancer risk behaviours originate in adolescence: a longitudinal analysis using ALSPAC, a UK birth cohort study. BMC Cancer. 2021;21(1):1–15. 33827470 10.1186/s12885-021-08098-8 PMC8028717

[18] Boyd A, Macleod J, Lawlor DA, Fraser A, Henderson J, Molloy L, Ness A, Ring S, DaveySmith G. Cohort profile: the ’children of the 90s’-the index offspring of the avon longitudinal study of parents and children. Int J Epidemiol. 2013;42(1):111–27. 10.1093/ije/dys064. 22507743 10.1093/ije/dys064 PMC3600618

[19] Fraser A, Macdonald-wallis C, Tilling K, et al. Cohort profile: the avon longitudinal study of parents and children: ALSPAC mothers cohort. Int J Epidemiol. 2013;42(1):97–110. 10.1093/ije/dys066. 22507742 10.1093/ije/dys066 PMC3600619

[20] Troy D, Russell A, Kidger J, Wright C. Childhood psychopathology mediates associations between childhood adversities and multiple health risk behaviours in adolescence: analysis using the ALSPAC birth cohort. J Child Psychol Psychiatry Allied Discip. 2021. 10.1111/jcpp.13379. 10.1111/jcpp.13379 PMC8532527 33619761

[21] Royston P, White IR. Multiple imputation by chained equations (MICE): implementation in Stata. J Stat Softw. 2011;45:1–20.

[22] StataCorp. Stata statistical software: release 15. 2017.

[23] White IR, Royston P, Wood AM. Multiple imputation using chained equations: issues and guidance for practice. Stat Med. 2011;30(4):377–99. 21225900 10.1002/sim.4067

[24] Kipping RR, Campbell RM, MacArthur GJ, Gunnell DJ, Hickman M. Multiple risk behaviour in adolescence. J Public Health (Bangkok). 2012;34(suppl_1):i1–2. 10.1093/pubmed/fdr122. 10.1093/pubmed/fdr122 22363025

[25] Champion KE, Mather M, Spring B, Kay-Lambkin F, Teesson M, Newton NC. Clustering of multiple risk behaviors among a sample of 18-year-old Australians and associations with mental health outcomes: a latent class analysis. Front Public Heal. 2018;6:1–11. 10.3389/fpubh.2018.00135. 10.3389/fpubh.2018.00135 PMC5949382 29868543

[26] Harakeh Z, De Looze ME, Schrijvers CTM, van Dorsselaer SAFM, Vollebergh WAM. Individual and environmental predictors of health risk behaviours among Dutch adolescents: the HBSC study. Public Health. 2012;126(7):566–73. 10.1016/j.puhe.2012.04.006. 22607981 10.1016/j.puhe.2012.04.006

[27] Taylor A. The Impact of Parental Substance Misuse on Child Development.; 2013. https://www.cumbria.gov.uk/eLibrary/Content/Internet/537/6683/6684/4352993837.pdf.

[28] Ornelas IJ, Perreira KM, Ayala GX. Parental influences on adolescent physical activity: a longitudinal study. Int J Behav Nutr Phys Act. 2007;4(1):3. 10.1186/1479-5868-4-3. 17274822 10.1186/1479-5868-4-3 PMC1805507

[29] Lander L, Howsare J, Byrne M. The impact of substance use disorders on families and children: from theory to practice. Soc Work Public Health. 2013;28(3–4):194–205. 10.1080/19371918.2013.759005. 23731414 10.1080/19371918.2013.759005 PMC3725219

[30] Xerxa Y, Rescorla LA, Serdarevic F, et al. The complex role of parental separation in the association between family conflict and child problem behavior. J Clin Child Adolesc Psychol. 2020;49(1):79–93. 10.1080/15374416.2018.1520118. 30657708 10.1080/15374416.2018.1520118

[31] DiClemente RJ, Wingood GM, Crosby R, et al. Parental monitoring: association with adolescents’ risk behaviors. Pediatrics. 2001;107(6):1363–8. 11389258 10.1542/peds.107.6.1363

[32] Bellis MA, Hughes K, Leckenby N, Perkins C, Lowey H. National household survey of adverse childhood experiences and their relationship with resilience to health-harming behaviors in England. BMC Med. 2014;12(1):72. 10.1186/1741-7015-12-72. 24886026 10.1186/1741-7015-12-72 PMC4234527

[33] Garrido EF, Weiler LM, Taussig HN. Adverse childhood experiences and health-risk behaviors in vulnerable early adolescents. J Early Adolesc. 2018;38(5):661–80. 10.1177/0272431616687671. 29861530 10.1177/0272431616687671 PMC5976451

[34] Espeleta HC, Brett EI, Ridings LE, Leavens ELS, Mullins LL. Childhood adversity and adult health-risk behaviors: examining the roles of emotion dysregulation and urgency. Child Abuse Negl. 2018;82:92–101. 10.1016/j.chiabu.2018.05.027. 29879586 10.1016/j.chiabu.2018.05.027

[35] Kelly-Irving M, Lepage B, Dedieu D, et al. Childhood adversity as a risk for cancer: findings from the 1958 British birth cohort study. BMC Public Health. 2013;13(1):767. 10.1186/1471-2458-13-767. 23957659 10.1186/1471-2458-13-767 PMC3765119

[36] Houtepen LC, Heron J, Suderman MJ, Tilling K, Howe LD. Adverse childhood experiences in the children of the Avon Longitudinal Study of Parents and Children (ALSPAC). Wellcome open Res. 2018;3:106. 10.12688/wellcomeopenres.14716.1. 30569020 10.12688/wellcomeopenres.14716.1 PMC6281007

[37] Hughes RA, Heron J, Sterne JAC, Tilling K. Accounting for missing data in statistical analyses: multiple imputation is not always the answer. Int J Epidemiol. 2019;48(4):1294–304. 30879056 10.1093/ije/dyz032 PMC6693809

