# Supplementary material for: Exploring the associations between adverse childhood experiences (ACEs) and adolescent cancer risk behaviours in the ALSPAC cohort
Source: BMC Public Health. 2024 Jan 5;24:95. doi: 10.1186/s12889-023-17516-4 (PMC10768201; doi:10.1186/s12889-023-17516-4)
Supplement: Supplementary file 1 — Additional file 1. [file 12889_2023_17516_MOESM1_ESM.docx]

**EXPLORING THE ASSOCIATIONS BETWEEN ADVERSE CHILDHOOD EXPERIENCES (ACES) AND ADOLESCENT CANCER RISK BEHAVIOURS IN THE ALSPAC COHORT**

*Paul Okediji, David Troy, Jon Heron, Ruth R Kipping, Richard M Martin, Caroline Wright*

**SUPPLEMENTARY MATERIAL**

**SECTION A. DERIVATION OF MODEL VARIABLES**

**SA-1. Exposure Variables**

Data on childhood adversity were collected via 288 questions asked over 27 data collection points. Mothers and partners responded about their child’s exposure to eight adverse childhood experiences (ACEs) and child bullying was based on responses obtained directly from the child. The definitions for each of the adversities included in this study is summarized in Table S1 below and based on definitions used in previous studies.^1,2^

As shown in Table S1, there are a number of questions contributing to each adverse childhood experience, ranging from 6 to 62 questions. Each of these questions/variables were dichotomised to a binary yes/no based on a pre-set criteria as defined by Houtepen et al.^1^ A child is considered to have experienced a particular type of adverse childhood experience if there is at least one value ‘yes’ for any of the dichotomised variables within each type of adverse childhood experience by the time the child is 9 years old. For example, there are seven questions relating to sexual abuse as indicated in Table S1. If the child records at least one ‘yes’ among the seven variables, the child is considered to have experienced sexual abuse.

**Table S1. Adversity definitions**

| **Adversity** | **Definition** | **Number of questions** | **Age range of questions asked** |
| --- | --- | --- | --- |
| Sexual abuse | Whether the child was sexually abused | 7 | 18mn – 9yr |
| Physical abuse | Whether parents have been physically cruel to child | 31 | 8wk – 9yr |
| Emotional abuse | Whether parents have been emotionally cruel to the child | 32 | 8mn – 9yr |
| Parent substance use | Whether mother/partner uses cannabis daily or any other drugs; or self-reported problematic use of alcohol, and saw a doctor because of it | 62 | 8wk – 9yr |
| Parent mental health problems or suicide | Scores >12 on the Edinburgh Postnatal Depression Scale and medication, presence of schizophrenia, bulimia, anorexia or attempted suicide | 57 | 8wk – 9yr |
| Violence between parents | Parent experienced physical cruelty from partner, or displayed (specific types) of violence towards partner | 43 | 8wk – 9yr |
| Parental separation | Parents divorced or separated | 32 | 8wk – 9yr |
| Bullying | Child has been bullied | 6 | 8yr – 8.5yr |
| Parent criminal conviction | Parent convicted of criminal offence | 18 | 8wk – 9yr |

**SA-2. Outcome Measure – Cancer Risk Behaviour**

Data were collected on adolescents’ engagement in five health risk behaviours that have been demonstrated to have causal relationships with cancer. Obesity is not a behaviour in itself but has been classified as one in many studies – partly for ease of description and also because it has underlying behavioural correlates such as inadequate physical activity and excess energy intake.^3^ Both obesity and physical inactivity have also been documented to have independent unconfounded causal relationships with cancer development.^4^

Risk thresholds used for each of these behaviours at ages 11, 14, 16, and 18 are as previously documented in a previous paper *(Table S2)*.^4^ Each CRB is treated as a binary measure and the total amount of CRBs each adolescent engages in was summarized using the following formula:

The area under the curve (AUC) formula:

$$\frac{Y_{1}+Y_{2}}{2}\left( t_{2}-t_{1} \right)+ \frac{Y_{2}+Y_{3}}{2}\left( t_{3}-t_{2} \right)+\frac{Y_{3}+Y_{4}}{2}\left( t_{4}-t_{3} \right)$$

Here Y_x_ represents the number of risk behaviours adolescents reported at each time, t_x_, and does not incorporate the behaviours engaged in at earlier ages As illustrated in Fig. S1 overleaf, for a participant with one CRB (Y_1_ = 1) at T1 (age 11), two CRBs (Y_2_ = 2) at T2 (age 14), three CRBs (Y_3_ = 3) at T3 (age 16), and five (Y_4_ = 5) at T4 (age 18), the total CRB engagement (area under the curve) is calculated as:

AUC = $\frac{1+2}{2}\left( 14-11 \right)+ \frac{2+3}{2}\left( 16-14 \right)+\frac{3+5}{2}\left( 18-16 \right)$ = 17.5

The area under the curve provides a measure of engagement in CRBs across all four age points on numeric-continuous scale, which makes it possible to rank all participants by their total CRB level. We note that this approach does not take the timing of the CRB into consideration which might be important as earlier engagement in certain CRBs may be more detrimental in terms of cancer development than later involvement, nor the nature of the specific CRB reported. Neither does this approach permit the behaviours to influence the scale differentially. As we state in our previous publication: “given the highly differential risks associated with these behaviours, at different levels, and for different cancers, each risk has an equal weighting, as it would not be possible to accurately weight the risk behaviours differently” (Wright et al.^4^).

**Figure S1. Summarizing engagement in cancer risk behaviour in adolescence as each participant’s Area Under the Curve (AUC) using self-reports at 4 waves**

**
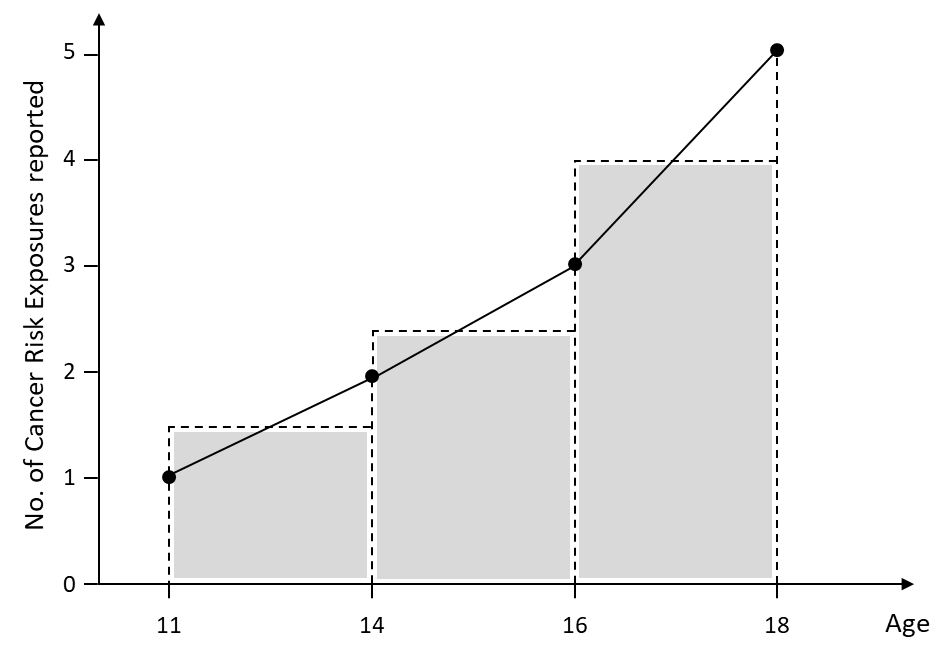
**

**Table S2. Risk thresholds for adolescent cancer risk behaviours. Adapted from Wright et al.^4^**

| **Cancer risk behaviour** | **11 years** | **14 years** | **16 years** | **18 years** |
| --- | --- | --- | --- | --- |
| **Tobacco smoking** | Adolescent has ever smoked | Adolescent has smoked cigarettes in past 6 months | Adolescent smokes every week | Adolescent smokes every week |
| **Alcohol consumption** | Adolescent has had a whole drink before age 12 years | Adolescent has had whole drink in past 6 months | Adolescent has had 6 or more whole drinks in past 30 days | Adolescent consumes alcohol ≥2-3 times per week or has hazardous alcohol consumption |
| **Unsafe sex** | Adolescent has had penetrative sex without the use of a condom on the last occasion they had sex in the past year | Adolescent has had penetrative sex without the use of a condom on the last occasion they had sex in the past year | Adolescent has had penetrative sex without the use of a condom on the last occasion they had sex in the past year | Adolescent has had penetrative sex without the use of a condom on the last occasion they had sex in the past year |
| **Physical inactivity** | Adolescent has participated in vigorous physical activity 1-3 times a week or less (parent report) | Adolescent typically exercises <5 times per week (self-report) or has participated in vigorous physical activity 1-3 times a week or less (parent report) | Adolescent typically exercises <5 times per week (self-report) or has participated in vigorous physical activity 1-3 times a week or less (parent report) | Adolescent typically exercises <5 times per week (self-report) or has participated in vigorous physical activity 1-3 times a week or less (parent report) |
| **Obesity** | Adolescent has a UK 1990 BMI population reference ≥95th percentile | Adolescent has a UK 1990 BMI population reference ≥95th percentile | Adolescent has a UK 1990 BMI population reference ≥95th percentile | Adolescent has a UK 1990 BMI population reference ≥95th percentile |

**SECTION B. SAMPLE DERIVATION / MISSING DATA CONSIDERATIONS**

Given that both the exposure and outcome were composite in nature, drawing together many variables from multiple waves of data collection, we made a pragmatic decision in order to reduce the computational burden of the imputation step. We initially defined our analysis sample based on the availability of CRB data in adolescence. To be included in the imputation sample (n = 7,358) a participant had to have provided information on a complete set of CRBs at at least one wave (11, 14, 16 OR 18 years). This allowed us to impute total CRB at the wave-level rather than impute as individual constituent parts shown in Table S2. Within this sample, we imputed any missing wave-level CRB-scores, ACEs (imputed individually and combined into the total score using the passive () command), and incomplete confounder information.

The complete case sample (n = 1,368) is small given the wealth of data required to derive the various components of the model. The flowchart in Figure S2 overleaf indicates that 6,598 individuals were excluded due to insufficient CRB information and a further 5,990 were excluded from the complete case analysis but incorporated into the imputation step.

**Figure S2. Participant flowchart.**


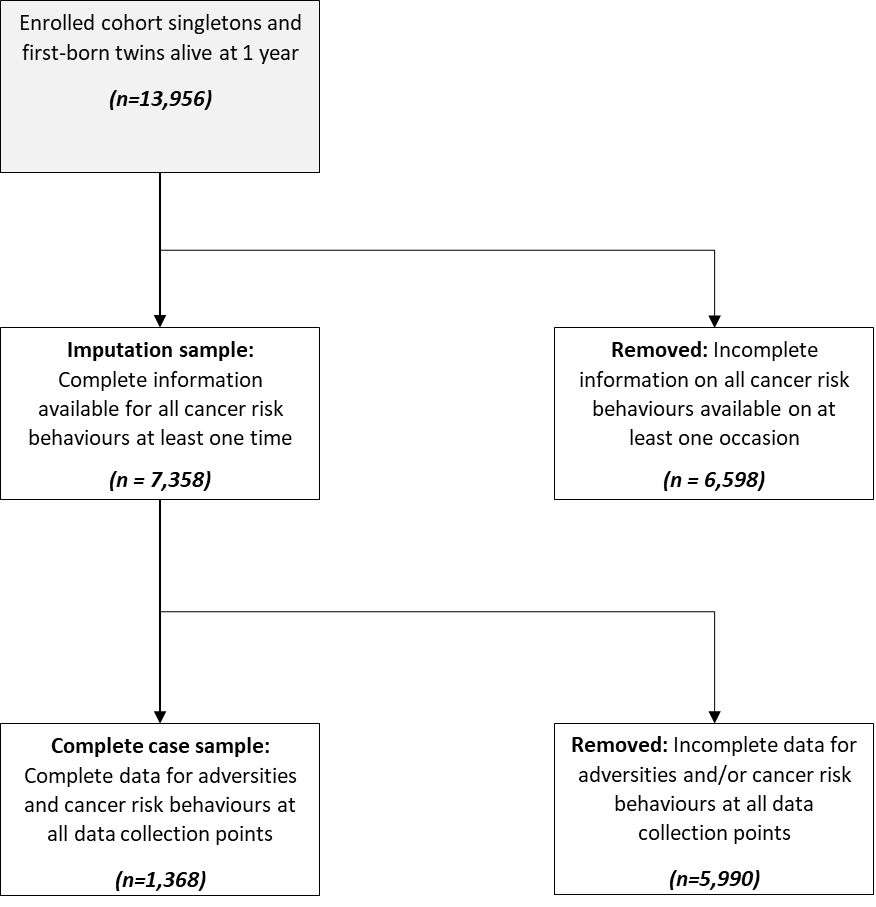


**SB-1. Degree of Missingness in the Dataset**

Not all the variables in the dataset had complete information represented for all the participants *(Table S3)*. The extent of missingness varies from variable to variable with only participant sex, birthweight, gestational age, and age of mother having 100% completeness. Regarding the outcome measure (combined CRB variable), 86.0% of the participants did not have data for all the CRBs investigated across all four timepoints of measurement.

**Table S3. Completeness of information for key variables used in the study.**

| **Variable** | **Missing** | **Total** | **% missing** |
| --- | --- | --- | --- |
| Sex | 0 | 13,956 | 0 |
| Birthweight | 0 | 13,956 | 0 |
| Age of mother at delivery | 0 | 13,956 | 0 |
| Housing tenure | 1,402 | 13,956 | 10.1 |
| Marital status | 889 | 13,956 | 6.4 |
| Parental social class | 2,462 | 13,956 | 17.6 |
| Gestational age | 0 | 13,956 | 0 |
| Household income | 7,766 | 13,956 | 55.7 |
| Mothers’ highest level of education | 1,559 | 13,956 | 11.2 |
| Combined adolescent CRB measure *(outcome)* | 12,007 | 13,956 | 86.0 |
| Childhood adversities *(exposure)* |  |  |  |
| Physical abuse | 5,910 | 13,956 | 42.4 |
| Sexual abuse | 4,424 | 13,956 | 31.7 |
| Emotional abuse | 6,810 | 13,956 | 48.8 |
| Bullying | 7,311 | 13,956 | 52.4 |
| Violence between parents | 7,606 | 13,956 | 54.5 |
| Parental substance use | 6,361 | 13,956 | 45.6 |
| Parents’ mental health problems or suicide | 6,209 | 13,956 | 44.5 |
| Parent conviction | 5,660 | 13,956 | 40.6 |
| Parental separation | 6,481 | 13,956 | 46.4 |
| Combined adversities measure | 9,701 | 13,956 | 69.5 |

**SB-2. Comparison of Full Dataset with Imputation and Complete Case Samples**

The first step in using the results of the analysis was to review the spread of complete data across the different datasets which included the full overall ALSPAC data set of 13,956 participants and the imputation and complete case samples of 7,358 and 1,368 respectively. The intent was to see if the results in the complete case samples for each exposure measure is influenced by any of the variables.

As presented in Table S4 below, participants in the final complete case sample are more likely to be females (59.1% in complete case sample vs. 48.4% in original full dataset), professionals/managers/technicians (proportion increased relative to those in the lower social classes who had a slightly lower chance of being included in the complete case sample), earn incomes in the middle-high or high quintiles (those with lower household incomes were less likely to be included in the complete case sample), live in mortgaged or owned property, and have education above O-level. This might present a problem in terms of bias in the final analysis if the outcome measure (cancer risk behaviours at ages 11, 14, 16, and 18 years) was conditionally dependent on the inclusion or exclusion of the participants in the analysis. However, by adjusting for all potential confounders such as sex, social class, mother's education, household income, housing tenure, and mother's age in the linear regression models, it is expected that any residual bias related to the selection of the sample and the final outcome will be minimal and not significantly influence the reliability of the findings.

**Table S4. Comparison of full ALSPAC data set with the imputation and complete case samples (observed data only)**

| **Variable** | **Overall ALSPAC sample** | | **Imputation sample** | | **Complete case sample** | |
| --- | --- | --- | --- | --- | --- | --- |
|  | **N** | **N (%)** | **N** | **N (%)** | **N** | **N (%)** |
| **Sex** |  |  |  |  |  |  |
| Female | 13,956 | 7205 (51.6%) | 7,358 | 3603 (49.0%) | 1,368 | 809 (59.1%) |
| Male |  | 6751 (48.4%) |  | 3755 (51.0%) |  | 559 (40.9%) |
| **Age of mother at delivery** |  |  |  |  |  |  |
| <18 years | 13,956 | 184 (1.3%) | 7,358 | 26 (0.4%) | 1,368 | 2 (0.2%) |
| 18 – 25 years |  | 4105 (29.4%) |  | 1479 (20.1%) |  | 197 (14.4%) |
| 26 – 35 years |  | 8701 (62.3%) |  | 5252 (71.4%) |  | 1027 (75.1%) |
| >35 years |  | 966 (6.9%) |  | 601 (8.2%) |  | 142 (10.4%) |
| **Parents’ highest social class** |  |  |  |  |  |  |
| Professional | 11,494 | 1525 (13.3%) | 5,203 | 977 (18.8%) | 1,368 | 326 (23.8%) |
| Managerial/technical |  | 4802 (41.8%) |  | 2454 (47.2%) |  | 656 (48.0%) |
| Skilled (non-manual) |  | 2928 (25.5%) |  | 1175 (22.6%) |  | 282 (20.6%) |
| Skilled (manual), part or unskilled |  | 2231 (19.4%) |  | 597 (11.5%) |  | 104 (7.6%) |
| **Mother’s education** |  |  |  |  |  |  |
| < O-level | 12,397 | 3726 (30.1%) | 7,246 | 1537 (21.2%) | 1,368 | 151 (11.0%) |
| O-level |  | 4290 (34.6%) |  | 2574 (35.5%) |  | 441 (32.2%) |
| > O-level |  | 4381 (35.3%) |  | 3135 (43.3%) |  | 776 (56.7%) |
| **Household income in quintiles** |  |  |  |  |  |  |
| High | 6,190 | 1455 (23.5%) | 5,190 | 1341 (25.8%) | 1,368 | 396 (29.0%) |
| Middle high |  | 1354 (21.9%) |  | 1203 (23.2%) |  | 366 (26.8%) |
| Middle |  | 1262 (20.4%) |  | 1075 (20.7%) |  | 302 (22.1%) |
| Middle low |  | 1145 (18.5%) |  | 915 (17.6%) |  | 198 (14.5%) |
| Low |  | 974 (15.7%) |  | 656 (12.6%) |  | 106 (7.8%) |
| **Home ownership status** |  |  |  |  |  |  |
| Mortgaged/owned property | 12,554 | 9540 (76.0%) | 7,225 | 6079 (84.1%) | 1,368 | 1247 (91.2%) |
| Privately rented property |  | 933 (7.4%) |  | 605 (8.4%) |  | 44 (3.2%) |
| Subsidized rental property |  | 2081 (16.6%) |  | 541 (7.5%) |  | 77 (5.6%) |

**Table S5. Linear regression analyses showing associations between childhood adversity and adolescent multiple cancer risk behaviours *(N = 1,368; complete case sample)***

| **Childhood adversity variable** | **Univariable  regression models** | | | **Multivariable*  regression models** | | |
| --- | --- | --- | --- | --- | --- | --- |
|  | **β** | **95% CI** | **p** | **β** | **95% CI** | **p** |
| Adverse childhood experience total score (per additional ACE) | 0.46 | 0.27 ‒ 0.64 | <0.001 | 0.45 | 0.26 ‒ 0.63 | <0.001 |
| Physical abuse | 0.54 | -0.37 ‒ 1.44 | 0.244 | 0.69 | -0.21 ‒ 1.59 | 0.131 |
| Sexual abuse | 2.50 | -1.17 ‒ 6.16 | 0.181 | 2.09 | -1.57 ‒ 5.75 | 0.263 |
| Emotional abuse | 0.61 | -0.01 ‒ 1.24 | 0.055 | 0.63 | 0.01 ‒ 1.26 | 0.046 |
| Bullying | 0.77 | 0.04 ‒ 1.51 | 0.039 | 0.71 | -0.03 ‒ 1.44 | 0.059 |
| Violence between parents | 0.54 | -0.07 ‒ 1.15 | 0.084 | 0.56 | -0.05 ‒ 1.17 | 0.074 |
| Substance use | 1.10 | 0.22 ‒ 1.97 | 0.014 | 1.08 | 0.20 ‒ 1.95 | 0.016 |
| Mental health problems | 0.66 | 0.19 ‒ 1.14 | 0.006 | 0.63 | 0.15 ‒ 1.10 | 0.010 |
| Parent conviction | 0.97 | 0.00 ‒ 1.93 | 0.049 | 0.91 | -0.05 ‒ 1.87 | 0.064 |
| Parental separation | 1.26 | 0.55 ‒ 1.98 | 0.001 | 1.12 | 0.40 ‒ 1.85 | 0.002 |

* Estimates controlled for sex, social class, mother's education, household income, housing tenure, and mother's age.

β indicates that each additional adverse childhood experience leads to a unit increase in the number of cancer risk behaviours

Results based on complete case sample.

**SECTION C. DISTRIBUTION OF STUDY POPULATION BY PREVALENCE OF CANCER RISK BEHAVIOURS**

Across all five CRBs investigated, there was a general increase in prevalence as the adolescents grew older except for obesity *(Fig. S3)*. The rate of increase over time was found to be higher with physical inactivity and unsafe sex. At age 11, 64.3% of the adolescents were physically inactive, increasing to 86.3% by age 18 years (a 34.2% increase over seven years of life). Similarly, the proportion of adolescents engaging in unsafe sex at age 11 was zero. This increased steadily over the following years to as high as 27.0% by age 18 years. The prevalence of alcohol consumption rose gradually from 39.0% at age 11 to 45.3% at age 16 and dropped to 42.7% at age 18. The prevalence of obesity remained fairly the same between ages 11 and 18 with a slight drop from 12.5% to 10.2% by age 14 and a slight increase to 11.7% by age 18.

With regards to tobacco smoking, the prevalence almost doubled from 7.7% at age 14 years to 14.9% at age 18 years. While this appears to be a huge increase, recent evidence suggests that the prevalence of tobacco smoking among adolescents in the UK has been steadily declining.^5^ On the other hand, the use of e-cigarettes is increasing among children and young people.^5,6^ Although the relationship between the use of e-cigarettes and cancer is not clear, there are concerns that e-cigarette use among adolescents may be a gateway to tobacco smoking with its attendant implications on cancer risk.^5^

**Figure S3.** Changing prevalence of each cancer risk behaviour during adolescence (*N = 1,368, complete case sample*)

**SECTION D. Mutual Adjustment for other ACEs**

Below we present the parameters from a final regression model in which all ACEs have been mutually adjusted. We stress that whilst observing these independent effects may be informative, the complex causal inter-relationships between ACEs means that some caution should be exercised.

There appeared to be a reduction in the beta-coefficients of the regression models adjusted for both confounders and other ACEs from what they were in the models adjusted for only confounders. In the confounder adjusted models, emotional abuse, substance use, violence between parents, mental health problems in parents and parental separation demonstrated strong evidence of association with cancer risk behaviours in adolescence (p < 0.05, Table S6). Among this set of confounder-adjusted models, parental substance use was the most strongly associated with cancer risk behaviour with a 0.64 unit increase in cancer risk behaviour (p = 0.001). In the model adjusted for both confounders and other ACEs, the beta coefficient had weakened to 0.48, attenuating the association between parental substance use and cancer risk behaviour. Similar attenuations in the association were noted across board for all the other ACEs.

Table S6. Association between each ACE and engagement in CRB both before and after mutual adjustment for other ACEs. (n = 7,358, imputed sample).

|  | **Confounder adjusted models** | | | **Models adjusted for confounders and mutually adjusted for other ACEs** | | |
| --- | --- | --- | --- | --- | --- | --- |
| **Childhood adversity** | **β** | **95% CI** | **p** | **β** | **95% CI** | **p** |
| Physical abuse | 0.36 | -0.09 ‒ 0.81 | 0.113 | -0.05 | -0.53 ‒ 0.44 | 0.851 |
| Sexual abuse | 0.74 | -0.88 ‒ 2.36 | 0.373 | 0.46 | -1.16 ‒ 2.08 | 0.577 |
| Emotional abuse | 0.51 | 0.20 ‒ 0.82 | 0.001 | 0.28 | -0.05 ‒ 0.62 | 0.098 |
| Bullying | 0.22 | -0.13 ‒ 0.58 | 0.220 | 0.19 | -0.17 ‒ 0.55 | 0.297 |
| Violence between parents | 0.38 | 0.08 ‒ 0.68 | 0.012 | 0.19 | -0.12 ‒ 0.50 | 0.236 |
| Substance use | 0.64 | 0.25 ‒ 1.03 | 0.001 | 0.48 | 0.08 ‒ 0.87 | 0.017 |
| Mental health problems | 0.42 | 0.18 ‒ 0.65 | <0.001 | 0.27 | 0.03 ‒ 0.51 | 0.030 |
| Parent conviction | 0.28 | -0.18 ‒ 0.74 | 0.232 | 0.10 | -0.36 ‒ 0.56 | 0.676 |
| Parental separation | 0.56 | 0.27 ‒ 0.86 | <0.001 | 0.37 | 0.07 ‒ 0.68 | 0.017 |

**REFERENCES FOR SUPPLEMENTARY MATERIAL**

1. Houtepen LC, Heron J, Suderman MJ, Tilling K, Howe LD. Adverse childhood experiences in the children of the Avon Longitudinal Study of Parents and Children (ALSPAC). *Wellcome open Res*. 2018;3:106. doi:10.12688/wellcomeopenres.14716.1

2. Russell AE, Heron J, Gunnell D, et al. Pathways between early-life adversity and adolescent self-harm: the mediating role of inflammation in the Avon Longitudinal Study of Parents and Children. *J Child Psychol Psychiatry*. 2019;60(10):1094-1103. doi:10.1111/jcpp.13100

3. Hausdorf K, Eakin E, Whiteman D, Rogers C, Aitken J, Newman B. Prevalence and correlates of multiple cancer risk behaviors in an Australian population-based survey: results from the Queensland Cancer Risk Study. *Cancer Causes Control*. 2008;19(10):1339-1347. doi:10.1007/s10552-008-9205-y

4. Wright C, Heron J, Kipping R, Hickman M, Campbell R, Martin RM. Young adult cancer risk behaviours originate in adolescence: a longitudinal analysis using ALSPAC, a UK birth cohort study. *BMC Cancer*. 2021;21(1):1-15.

5. Williams PJ, Cheeseman H, Arnott D, Bunce L, Hopkinson NS, Laverty AA. Use of tobacco and e-cigarettes among youth in Great Britain in 2022: Analysis of a cross-sectional survey. *Tob Induc Dis*. 2023;21:5. doi:10.18332/tid/156459

6. Pinho-Gomes A-C, Santos JA, Jones A, Thout SR, Pettigrew S. E-cigarette attitudes and behaviours amongst 15-30-year-olds in the UK. *J Public Health (Bangkok)*. 2023:fdad138. doi:10.1093/pubmed/fdad138
